# A Challenging Case of Carcinoid Crisis in a Patient With Neuroendocrine Tumor

**DOI:** 10.7759/cureus.15626

**Published:** 2021-06-13

**Authors:** Mohammed Mahdi, Muhammet Ozer, Muhammad Tahseen

**Affiliations:** 1 Internal Medicine, Capital Health System, Trenton, USA; 2 Critical Care Medicine, Capital Health System, Trenton, USA

**Keywords:** carcinoid crisis, neuroendocrine tumor, octreotide, hypotension, shock

## Abstract

The critical state of circulatory collapse and hypoperfusion that results in end-organ damage has been called shock. Carcinoid crisis is a rare cause of shock, which is difficult to identify in the presence of infection. It occurs due to the release of vasoactive amines into the systemic circulation following an inciting event. In the presence of a neuroendocrine tumor, carcinoid crisis should be suspected in case of resistant shock. Herein, we report a rare case of carcinoid crisis, in which a quick response to the somatostatin analog therapy, octreotide, was helpful to confirm the diagnosis.

## Introduction

Neuroendocrine tumors (NETs) are malignant neoplasms of enterochromaffin cells and/or gastrin cells that present predominantly in the gastroenteropancreatic system [1]. Traditionally they are classified based on the site of origin: foregut (stomach, duodenum, lungs, and pancreas), midgut (distal duodenum through hepatic flexure of ascending colon), and hindgut (transverse colon through rectum) [1]. NETs represent the second most common prevalent gastrointestinal cancers with a prevalence of 171,321 and an annual incidence rate of 6.98 per 100,000 as per the Surveillance, Epidemiology, and End Results (SEER) program of the National Cancer Institute [2].

Carcinoid syndrome (CS) is characterized by flushing, diarrhea, hypotension, tachycardia, telangiectasia, and in advanced stage valvular heart disease [3]. CS is caused by the largely secreted vasoactive amines directly into the systemic circulation bypassing the hepatic metabolism, which usually happens in extensive liver metastasis and bronchial carcinoids [4,5]. While serotonin (5-hydroxytryptamine) is considered to be the primary biomarker associated with CS, other vasoactive amines like prostaglandins, tachykinins, bradykinins, and histamine are also identified [5,6]. Here, we present a complicated case of circulatory collapse due to carcinoid crisis, etiology of which remained unclear and perhaps multifactorial.

## Case presentation

A 37-year-old African American male with past medical history of metastatic gastrointestinal neuroendocrine tumor (NET) and hemodialysis-dependent end-stage renal disease (ESRD) presented with worsening generalized abdominal pain and new onset hypotension. 

Three months ago, he was diagnosed with metastatic NET based on percutaneous liver biopsy. He received induction chemotherapy of carboplatin and etoposide along with programmed death ligand-1 (PD-L1) inhibitor atezolizumab. A month later, he started to have unbearable abdominal pain and diagnosed with intestinal mechanical obstruction due to tumor. He underwent explorative laparotomy with right hemicolectomy and ileostomy placement. Subsequently, he suffered a surgical wound infection that required necrotic tissue debridement and broad-spectrum antibiotic therapy. He improved and was discharged to a rehabilitation facility, where he spent two weeks and returned back for the current presentation. 

Physical examination was remarkable for depressed mood, hair loss, dark and coarse skin with freckles. His blood pressure was 77/50 mmHg, heart rate of 125 bpm, respiratory rate of 19 per minute, oxygen saturation of 98% on ambient air, and temperature of 36.6 Celsius. There was diffuse abdominal tenderness but clean and healing wounds. The Ileostomy bag contained a yellow-colored semi-formed stool. There was a right chest wall infusion port and left atrio-venous fistula without signs of infection. There was no murmur or S3 on heart auscultation. Jugular veins were non-distended and legs were normothermic and non-edematous. Lung auscultation was normal breath sounds without crackles or wheezes. neurological examination was unremarkable.

Investigations

The laboratory results are summarized in Table 1. Blood cultures were unyielding from four different sets. The wound tissue culture from the past wound infection grew *Escherichia coli* and vancomycin-susceptible *Enterococcus faecium*. 

**Table 1:** Laboratory results at presentation

Variable	Value (Reference range)
White blood count	11.1 x10^3^ (4-10 x10^3^ cells/ul)
Hemoglobin	9.2 (11.2-15.7 g/dl)
Platelets	294 x10^3^ (150-400 x10^3^ cells/ul)
Neutrophils	79% (35-70%)
Lymphocyte	12% (20-53%)
Bands	0 (0-8%)
Total protein	8.7 (6.5-8.5 g/dl)
Albumin	4 (3.5 – 5.0 g/dl)
Total bilirubin	2.7 (0.2 – 1.3 mg/dl)
Aspartate aminotransferase (AST)	88 (17 – 59 U/L)
Alanine transaminase (ALT)	34 (0 – 49 U/L)
Alkaline phosphatase (ALK-P)	230 (38 – 126 U/L)
Sodium	134 (137 – 145 mmol/L)
Potassium	4.4 (3.5 – 5.1 mmol/L)
Chloride	92 (98 – 107 mmol/L)
HCO3	27 (22 – 30 mmol/L)
Glucose	93 (70 -100 mg/dl)
Urea	24 (9 – 20 mg/dl)
Creatinine	5.41 (0.66 – 1.25 mg/dl)
Calcium	10.9 (8.6 – 10.3 mg/dl)
Troponin I	0.02 (0 – 0.034 ng/ml)
Lactic acid	2.7 (0.7 1.9 mmol/l)
Cortisol (a.m.)	47.5 (4.5 – 22.7 ug/dl)
Serotonin	<5 (21-321 ng/ml)
Chromogranin A	664.5 (0 – 101 ng/ml)

Transthoracic echocardiography revealed normal functioning ventricles without valvular abnormalities and a left ventricular ejection fraction of 55%. The repeated computed tomography (CT) scan of abdomen and pelvis from this admission was remarkable for stable multiple hepatic metastases and abdominal surgical defects.

**Figure 1:**
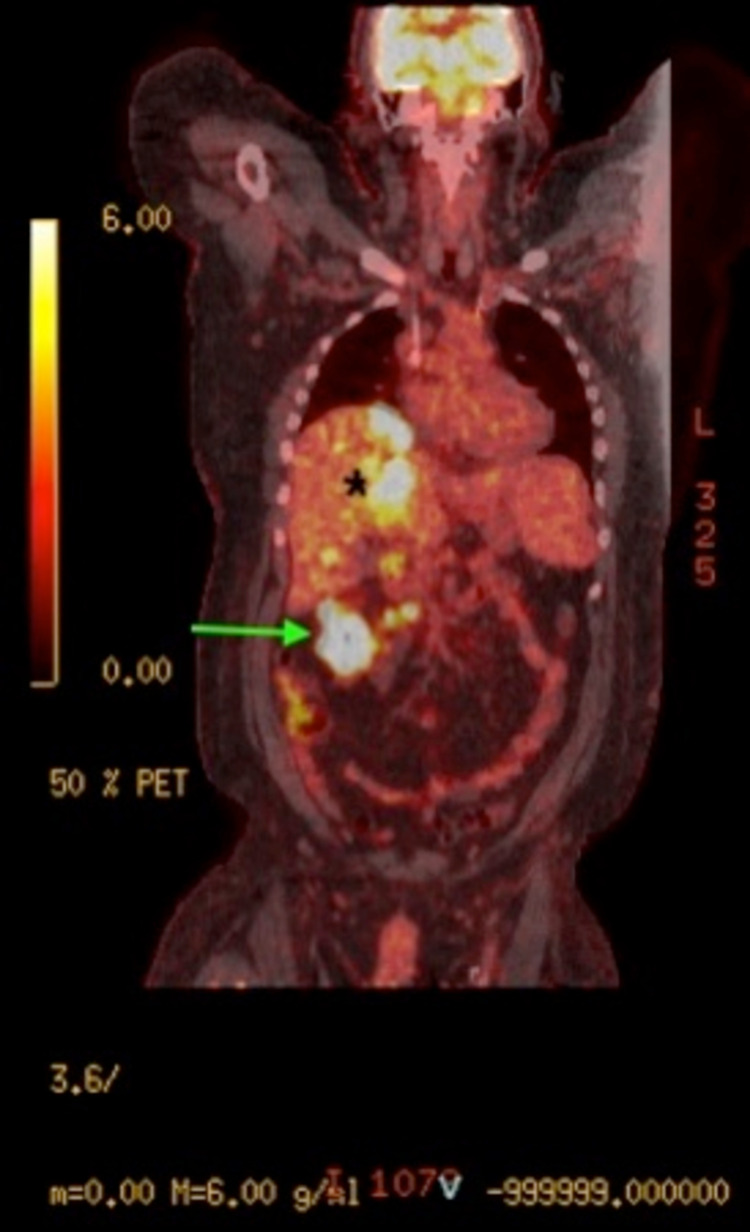
Coronal view positron emission tomography Shows primary transverse colon mass (arrow) with extensive metastasis to liver (star) and regional lymph nodes.

The patient’s historical workup included positron emission tomography and computed tomography (PET-CT) scan, which revealed primary transverse colon mass with extensive metastasis to liver and regional lymph nodes (Figure 1). The histopathological examination of liver biopsy was evident for high-grade neuroendocrine carcinoma with a Ki-67 proliferative rate of 60%. The immunohistochemical staining was positive for AE1/3, CK20, chromogranin, synaptophysin, CD56, CDX2, SATB2, TTF1, CK20, and CK19 and negative for p40, CK7, GATA3, and hepatocyte-specific antigen. 

Management

The patient was admitted to the intensive care unit. Intravenous antibiotic therapy with vancomycin, cefepime, and metronidazole was empirically initiated. The random vancomycin level was around 17 ug/ml. A titratable norepinephrine infusion therapy was started targeting a mean arterial pressure of equal or > 65 mmHg. Slight improvement in blood pressure was noticed; however, norepinephrine dose was increased gradually to a peak of 22 mcg per minute. Midodrine therapy with a dose of 10 mg three times daily did not make a remarkable difference. 

**Figure 2:**
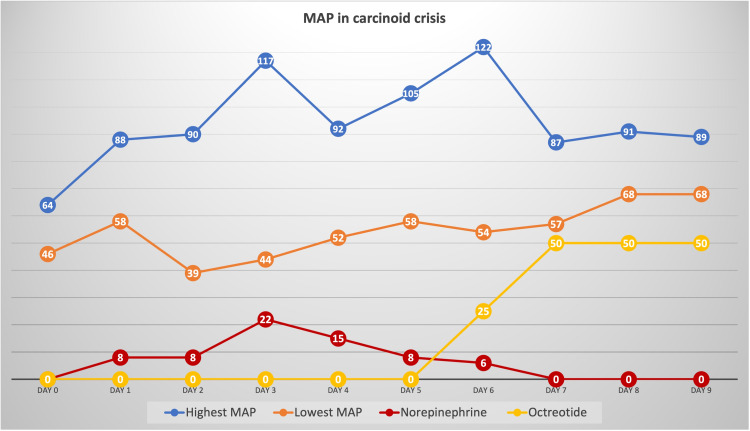
Response of blood pressure to octreotide therapy This chart shows the mean arterial pressure (MAP) response, with highest level in blue color and lowest level in orange color (all in mmHg), in relation to norepinephrine intravenous infusion (dose in mcg/minute) and octreotide therapy (dose in mcg/hour). Day zero is before norepinephrine initiation and Day 6 is when octreotide infusion started and norepinephrine was tapered off.

In keeping with the carcinoid crisis as a high likely diagnosis, urinary 5-Hydroxyindoleacetic Acid (5-HIAA) could not be checked as the patient was anuric. Instead, levels of serum serotonin and chromogranin-A were ordered. Only chromogranin-A level was elevated (Table 1). Intravenous octreotide infusion with a rate of 50 mcg/hour dose was initiated, and norepinephrine was gradually discontinued over 30 hours. The blood pressure improved quickly and dramatically and was maintained within normal limits without the need for vasopressors (Figure 2). Antibiotic therapy was switched to piperacillin-tazobactam, octreotide infusion was maintained at a rate of 50 mcg/hour and he was transferred to the medical floor in stable condition with blood pressure average of 115/80 mmHg and heart rate of 95 bpm.

Follow up

Due to poor performance status, end stage renal disease, and poor response to chemotherapy, the patient's goals of care were discussed and changed into comfort-measures-only and he was subsequently discharged to home. 

## Discussion

Carcinoid crisis is a rare and under-recognized syndrome of circulatory collapse caused by the sudden release of hormonal vasoactive amines after inciting factors. These could be any surgery, biopsy, chemotherapy, induction of general anesthesia, and even palpation of the tumor or its metastasis [6]. Carcinoid crisis can also manifest in stupor, confusion, bronchospasm, hyperthermia, and cardiac arrhythmias. This resembles septic shock, especially when a potential infection source is present, and thus a diagnosis of carcinoid crisis can be missed.

Interestingly, the administration of β-adrenergic agonists like epinephrine and norepinephrine was thought to potentiate the syndrome by inducing kallikrein enzymes that produce kinins from kininogens. Kinins cause profound vasomotor relaxation resulting in severe hypotension and flushing [7]. However, a recent study revealed that β-adrenergic agonist use was not associated with paradoxical hypotension, prolonged carcinoid crisis, or postoperative complications in patients with intraoperative carcinoid crisis [8]. There is an ongoing controversy about the role of preoperative parenteral octreotide in carcinoid crisis prevention with only weak supporting evidence for its use [9-11]. However, intravenous octreotide continuous infusion was deemed to be effective as a treatment [12].

The main differential diagnoses in this patient were septic shock, adrenal insufficiency, hypotension secondary to hemodialysis or ESRD, and carcinoid crisis. The refractoriness and persistence of hypotension despite several days of broad-spectrum antibiotics and vasopressors made septic shock an unlikely diagnosis. Adrenal insufficiency was possible due to long-standing dialysis-dependent ESRD; however, morning serum cortisol was appropriately elevated. The sudden development of hypotension and tachycardia was not typical of hypotension of dialysis-dependent ESRD, making the diagnosis less likely. 

Given the anuric ESRD, confirming active NET by measuring its biomarkers becomes difficult. Urinary 5-HIAA can not be checked due to the lack of urine. Serum serotonin can be falsely low and chromogranin-A can be falsely elevated [13]. In this case, a conclusion could not be drawn from the low serotonin and elevated chromogranin-A levels. However, the established diagnosis of hepatic metastasis of NET and failure to respond to vasopressors favored the diagnosis of carcinoid crisis, which was further supported by the quick and dramatic response to intravenous octreotide therapy.

While a specific triggering factor remained unclear, it can be due to (i) transcutaneous liver biopsy, (ii) upper and lower gastrointestinal endoscopy, (iii) induction chemotherapy with a PD-L1 inhibitor, (iv) mechanical bowel obstruction, (v) right hemicolectomy, (vi) abdominal surgical wound infection, or (vii) surgical wound debridement. Despite the fact that carcinoid crisis did not occur immediately after any of them, neither octreotide therapy was used as a preventive step. Finally, it is important to note that norepinephrine use was not associated with paradoxical hypotension as it was hypothesized.

## Conclusions

Carcinoid crisis is an important complication of neuro-endocrine tumors, which can masquerade as septic shock in cases where infection is a potential etiology. Refractoriness to vasopressors should increase the index of suspicion. Somatostatin analog therapy can be considered both diagnostic and therapeutic.

## References

[1] Williams ED, Sandler M (1963). The classification of carcinoid tumours. Lancet.

[2] Dasari A, Shen C, Halperin D (2017). Trends in the incidence, prevalence, and survival outcomes in patients with neuroendocrine tumors in the United States. JAMA Oncol.

[3] Mota JM, Sousa LG, Riechelmann RP (2016). Complications from carcinoid syndrome: review of the current evidence. Ecancermedicalscience.

[4] Caplin ME, Buscombe JR, Hilson AJ, Jones AL, Watkinson AF, Burroughs AK (1998). Carcinoid tumour. Lancet.

[5] Modlin IM, Kidd M, Latich I, Zikusoka MN, Shapiro MD (2005). Current status of gastrointestinal carcinoids. Gastroenterology.

[6] Lips CJ, Lentjes EG, Höppener JW (2003). The spectrum of carcinoid tumours and carcinoid syndromes. Ann Clin Biochem.

[7] Vaughan DJ, Brunner MD (1997). Anesthesia for patients with carcinoid syndrome. Int Anesthesiol Clin.

[8] Limbach KE, Condron ME, Bingham AE, Pommier SJ, Pommier RF (2019). Β-Adrenergic agonist administration is not associated with secondary carcinoid crisis in patients with carcinoid tumor. Am J Surg.

[9] Condron ME, Pommier SJ, Pommier RF (2016). Continuous infusion of octreotide combined with perioperative octreotide bolus does not prevent intraoperative carcinoid crisis. Surgery.

[10] Massimino K, Harrskog O, Pommier S, Pommier R (2013). Octreotide LAR and bolus octreotide are insufficient for preventing intraoperative complications in carcinoid patients. J Surg Oncol.

[11] Woltering EA, Wright AE, Stevens MA (2016). Development of effective prophylaxis against intraoperative carcinoid crisis. J Clin Anesth.

[12] Seymour N, Sawh SC (2013). Mega-dose intravenous octreotide for the treatment of carcinoid crisis: a systematic review. Can J Anaesth.

[13] Sansone A, Lauretta R, Vottari S, Chiefari A, Barnabei A, Romanelli F, Appetecchia M (2019). Specific and non-specific biomarkers in neuroendocrine gastroenteropancreatic tumors. Cancers (Basel).

